# Seismic Microzonation of Breginjski Kot (NW Slovenia) Based on Detailed Engineering Geological Mapping

**DOI:** 10.1155/2013/626854

**Published:** 2013-12-21

**Authors:** Jure Kokošin, Andrej Gosar

**Affiliations:** ^1^Sweco Norge AS, Dronningensgate 52/54, 8509 Narvik, Norway; ^2^Faculty of Natural Sciences and Engineering, University of Ljubljana, Aškerčeva 12, SI-1000 Ljubljana, Slovenia; ^3^Slovenian Environment Agency, Seismology and Geology Office, Dunajska 47, SI-1000 Ljubljana, Slovenia

## Abstract

Breginjski kot is among the most endangered seismic zones in Slovenia with the seismic hazard assessed to intensity IX MSK and the design ground acceleration of 0.250 g, both for 500-year return period. The most destructive was the 1976 Friuli Mw = 6.4 earthquake which had maximum intensity VIII-IX. Since the previous microzonation of the area was based solely on the basic geological map and did not include supplementary field research, we have performed a new soil classification of the area. First, a detailed engineering geological mapping in scale 1 : 5.000 was conducted. Mapped units were described in detail and some of them interpreted anew. Stiff sites are composed of hard to medium-hard rocks which were subjected to erosion mainly evoked by glacial and postglacial age. At that time a prominent topography was formed and different types of sediments were deposited in valleys by mass flows. A distinction between sediments and weathered rocks, their exact position, and thickness are of significant importance for microzonation. On the basis of geological mapping, a soil classification was carried out according to the Medvedev method (intensity increments) and the Eurocode 8 standard (soil factors) and two microzonation maps were prepared. The bulk of the studied area is covered by soft sediments and nine out of ten settlements are situated on them. The microzonation clearly points out the dependence of damage distribution in the case of 1976 Friuli earthquake to local site effects.

## 1. Introduction

**Figure 1:**
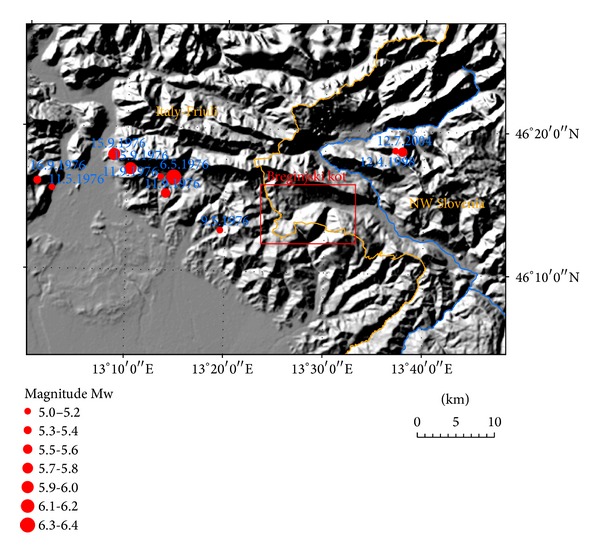
Seismicity (Mw ≥ 5.0) in Friuli and NW Slovenia with location of Breginjski kot study area.

The Breginjski kot (NW Slovenia, Figure 1) is located close to a seismically very active area of Friuli in NE Italy, but also the Julian Alps in Slovenia have recently experienced an increase of seismic activity. In this area, in fact, the seismic hazard in Slovenia is the highest. Most of numerous settlements in the area are built on soft sediments which can significantly enhance the ground shaking. The whole area was highly damaged during the Friuli 1976 Mw = 6.4 earthquake and its aftershock sequence. For a realistic assessment of site effects and earthquake hazard, a thorough seismic microzonation is therefore essential. On the other hand the existing microzonation of Breginjski kot [1] was based solely on the basic geological map of Slovenia in scale 1 : 100.000, without supplementary field investigations or subsurface information, thus making it fairly inaccurate. Therefore we have decided to perform a new microzonation study based on a detailed engineering geological mapping in scale 1 : 5.000. Such a map would enable soil classification according to two different approaches without using expensive geotechnical drilling or geophysical investigations. The seismic microzonation according to Eurocode 8 was based on a new European seismic standard [2] which has been applied in Slovenia from 2008. This standard defines the seismic hazard by the peak ground acceleration, whereas the site effects are expressed by the soil factor. The product of both values is used in the design of earthquake resistant buildings. The seismic microzonation according to the Medvedev method [3] denotes seismic hazard by increments of seismic intensity. Although the seismic intensity in Slovenia is not used for building design anymore, it is still important for seismological analyses and for the civil protection purposes.

## 2. Seismological Characteristics

The Breginjski kot is located in one of the three areas with the highest seismic hazard in Slovenia. This is mainly due to the proximity of the seismically very active area of Friuli located 10–30 km to the west in NE Italy. In this area the Mw = 6.4 Friuli earthquake occurred in May 1976. The seismic sequence that followed (Table 1, Figure 1) consisted of six events of Mw between 5.0 and 6.0 during the same year and one event of Mw = 5.3 that occurred in September 1977 [4, 5]. The intensity of the main shock in Breginj was VIII EMS-98, but the cumulative intensity which includes also damage from aftershocks reached IX EMS-98. The highest intensity in Slovenia from the main shock was VIII-IX in Robidišče [6]. A complete review of 1976 earthquake intensities in Breginjski kot settlements is summarized in Table 2.

For the NW Slovenia relatively weak rates of seismicity were observed before April 1998 when a Mw = 5.6 earthquake occurred in Krn Mountains, only 7 km NW from Kobarid [9, 10]. It was followed by a Mw = 5.2 event in July 2004 in the same epicentral area (Figure 1). Both earthquakes occurred on the NW-SE trending near-vertical dextral strike-slip Ravne fault [11] in the depth range 7.6–11 km. The intensity of the 1998 earthquake in Kobarid (located at eastern margin of the Breginjski kot) was VI-VII EMS-98 [7] and of the 2004 event VI EMS-98 [8].

NW Slovenia and Friuli region are located at the kinematic transition between E-W striking thrust faults of the Alpine system (Friuli earthquakes) and NW-SE striking strike-slip faults of the Dinarides system (Krn Mountains earthquakes) (Figure 1). The strongest earthquake ever recorded in the Alps-Dinarides junction area was the 1511 western Slovenia earthquake (Mw = 6.8); the exact location and mechanism of this event are still debated [12, 13] due to early occurrence and thus very little or no historical documents.

According to the old seismic hazard map of Slovenia for a 500-year return period showing expected intensities on the MSK scale [14], Breginjski kot is estimated to have IX MSK intensity. According to the new seismic hazard map for a 475-year return period [15], a design ground acceleration value for a rock site in Breginjski kot is 0.250 g. The important methodological difference between both maps is that intensities are assessed for a “medium” soil type, whereas design ground acceleration is assessed for a “rock” site according to Eurocode 8.

Accelerographs were installed in Kobarid after the main Friuli shock in 1976 and after the Krn Mountains earthquake in 1998. The strongest ground motion in the Friuli seismic sequence was recorded for the September 15, 1976 aftershock (Mw = 6.0, distance = 37 km) as 0.138 g peak ground acceleration [16]. For the July 12, 2004 Krn Mountain earthquake (Mw = 5.2, distance = 7 km) a peak ground acceleration of 0.152 g was recorded [17].

The previously existing microzonation of Breginjski kot [1] was based solely on the basic geological map in scale 1 : 100.000, without supplementary field research or subsurface geological and geophysical information, thus making it fairly inaccurate. It was prepared to be used together with the old seismic hazard map showing intensities on the MSK scale. This microzonation showed that the maximum expected intensity due to the effects of soft sediments can be increased by up to one intensity degree.

## 3. Engineering Geological Mapping

**Figure 2:**
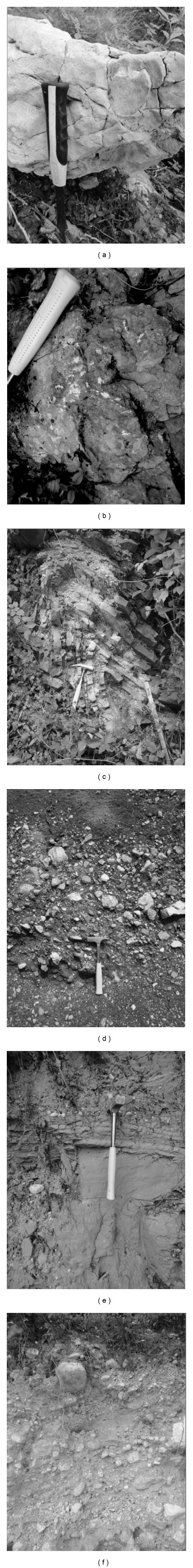
Typical lithologies according to the engineering-geological mapping: (a) Megalodontidae shell in Dachstein limestone in Robič, (b) limestone breccia interbedded with shalestone near Robidišče, (c) folds in flyschoid formation near Borjana, (d) heterogeneous composition of diamicton in Sedlo, (e) lacustrine chalk SW of Podbela, and (f) proluvium in Podbela.

**Figure 3:**
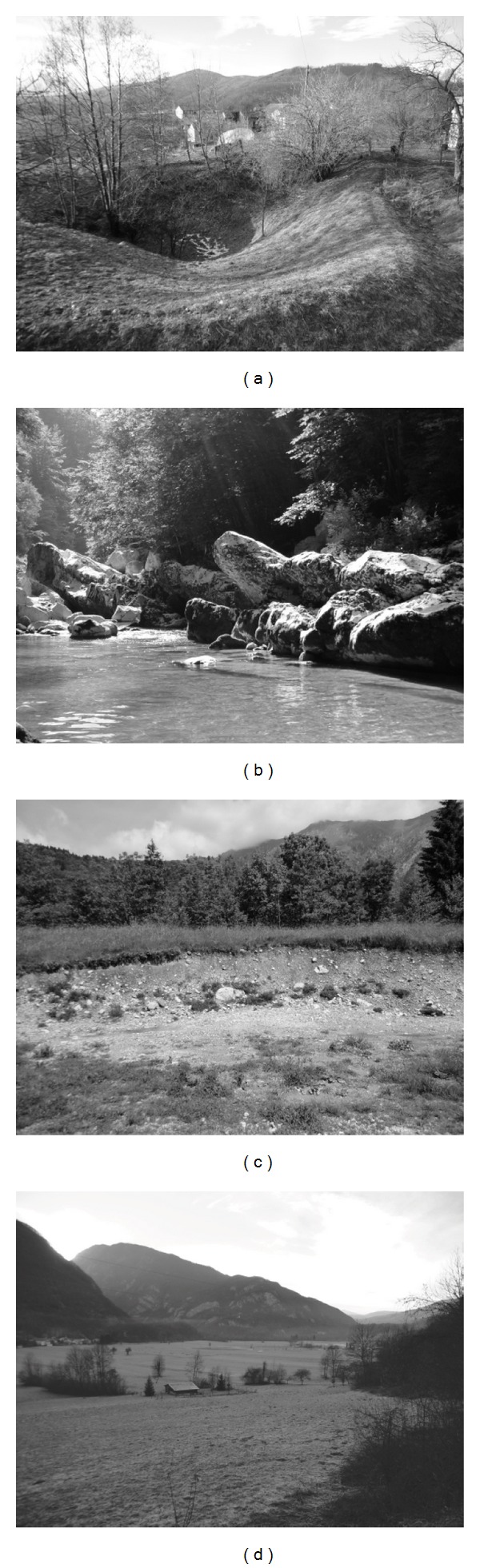
(a) Karst sinkhole in coral limestone represents a depositional place for significant amount of weathered material; Robidišče in the background. (b) Limestone breccia in Nadiža valley south of Logje is typically bedded. (c) Diamicton near Breginj, in the background Mt. Stol built of Dachstein limestone. (d) Alluvial fan sediments form gentle slopes above Kred polje, which is composed of fine grained alluvial sediments which promote to alluvial sediments near Nadiža River channel.

**Figure 4:**
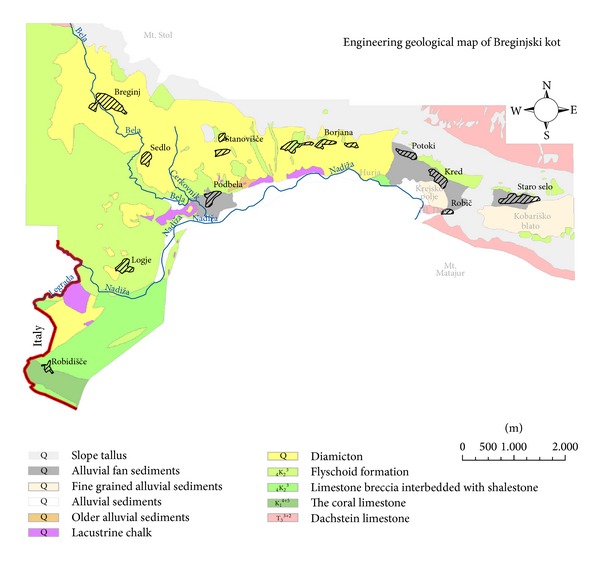
Engineering geological map of Breginjski kot.

A detailed engineering geological map of Breginjski kot in scale 1 : 5.000 was prepared by outcrop observing and geological boundaries tracking method. Since seismic microzonation was the final goal, the main focus of the field survey was to characterise local geological conditions in vicinity of settlements and other substantial infrastructure like roads, power lines, water collectors, and so forth. Consequently some less relevant lithological boundaries were summarised from the manuscript geological map in scale 1 : 25.000, which was the basis for published basic geological map in scale 1 : 100.000 [18]. Based on the mapping accomplished, rocks and sediments were classified by their characteristics determined in the field. Properties such as structure, dip, strength and weathered material thickness were defined for rocks, whereas granulation, roundness, and thickness were described for unconsolidated sediments. Mapped lithological units were compared with the Bovec basin, located 15 km to the NE in the Soča valley for which intensity increment microzonation was performed after the 1998 earthquake [19]. Lithologies described below (Figures 2 and 3) are arranged by age order and are presented on a detailed engineering geological map (Figure 4) of Breginjski kot. Their names and age classification mainly coincide with lithologies described in the explanatory text of the basic geological map of Slovenia [20].

*Dachstein limestone* (Figure 2(a)) from Late Triassic is the oldest formation in the studied area. It forms Mt. Stol, Mt. Matajur, and Der Hill west of Robič. Rock has a light grey colour and a crystalline texture. Dachstein limestone is characterized by Lofer development which consists of three parts. Cyclothem of B member contains stromatolites whereas cyclothem of C member contains well-preserved Megalodontidae shells. Cyclothem of A member consists of basal breccias but was not observed in the field. Weathered material thickness was estimated to be less than 0.5 m.

*The Coral limestone* of Early Cretaceous is situated in a small part of the examined area near Robidišče. Coral limestone is fine grained, massive and grey in colour. Outcrops are rare because they are overlain by significant thickness of weathered material. Hence it was distinguished from limestone breccias by numerous karst sinkholes which determine its morphology (Figure 3(a)). Moreover the weathered material thickness can be altered promptly as a consequence of a karst relief. On the slopes less than 1 m of weathered material is expected, whereas more than 5 m of weathered material is estimated for the flatland.

*Limestone breccia interbedded with shalestone* (Figure 2(b)) from Upper Cretaceous represents mainly the area from Napoleon Bridge west of Podbela to Robidišče (Figure 3(b)). Limestone breccias are of light grey or grey colour. Breccia is clast supported and composed of angular to subangular clasts which range from 2 mm to 1 m in size. Breccia formation is mostly bedded, rarely massive. If bedded, its thickness ranges from 10 cm to several meters in size, moreover brownish green shalestones are often interbedded. Formation generally dips to the north. On the slopes less than 1 m of weathered material is expected, whereas from 2 m to 3 m of weathered material is estimated for the flatland.

*Flyschoid formation* (Figure 2(c)) is the next formation of Upper Cretaceous and is situated mainly west of Breginj-Sedlo-Podbela line, while plenty outcrops are exposed in erosion windows of Mt. Stol and Mt. Matajur, especially in ravines with high-energy creeks. The Flyschoid formation is divided into series of rocks: shalestone, sandstone, and breccia, which are stratified and in sequence one after the other. Shalestone is dark grey and appears with thickness of beds ranging from 1 cm to 5 cm. Sandstone, which mostly consists of carbonate minerals, is grey with thickness of beds ranging from 5 cm to few ten centimetres. Breccia is grey to dark grey and consists of carbonate clasts ranging from 0.5 cm up to few meters. Coarse grained breccia is clast supported. Thickness of breccia beds ranges from 1 m to few ten meters. Flyschoid formation dips generally towards the north, moreover thin bedded series in particular are often found folded. Thickness of weathered material varies from 0.5 m to 1 m on the slopes and from 2 m to 3 m on the flatland.

Diverse Quaternary sediments in Breginjski kot are the result of postglacial era when water erosion and other transformational processes formed specific relief. Sediment deposits are mostly abundant on the slopes of Mt. Stol where most settlements are located.

*Diamicton* (Figure 2(d)) is an unlithified sediment which origins from glacial till mixed with other Quaternary sediments and transported by mass flow. It is deposited on the slope of Mt. Stol (Figure 3(c)), confined by Potoki to the east, a large area of flyschoid formation to the west and Nadiža River to the south. In addition some erosion patches can be found between Sedlo and Logje. According to the estimation on the field, diamicton is composed of more or less subrounded grains which are very poorly sorted. 70% of grains represent fine gravel, sand, and silt fractions, which ratio can vary significantly on studied locations. The remaining 30% is mainly gravel which ranges from 1 cm to 5 cm in size and boulders which ranges from 20 cm to several meters in size. Fragments consist of carbonate in general. Besides, there are many clastic fragments and diamictite ones, which are rather scarce. Glacial striations on fragments were not observed. Diamicton thickness varies significantly, for instance: along the Nadiža River only a few meters can be reached, in vicinity of flyschoid formation erosion windows it can vary between 5 m and 10 m, whereas away from ravines it can reach more than 20 m. The thickness depends on paleorelief and selective sedimentation processes, therefore a large amount of material has been deposited there. For instance in Cerkovnik creek flyschoid formation is not exposed, despite more than 20 m deep eroded ravine. According to ravine morphology, the biggest thickness is expected in the vicinity of Breginj.

*Diamictit* has the same origin as diamicton but it is lithified. It is not presented on the engineering-geological map, because it is scattered around the Diamicton area in small patches of about 10 m^2^ in size. It consists of around 70% of gravel and boulder sized clasts which are matrix supported. The latter is composed of silt, sand and fine gravel sized grains. Subrounded clasts are poorly sorted, those in the range from 0.5 cm to 5 cm prevail. Glacial striations on clasts are rarely preserved.

*Lacustrine chalk* (Figure 2(e)) is deposited in patches along broader zone of Nadiža River and in the largest portion besides Legrada creek. This sediment is characterised by laminated silt, grey in colour with a blue tinge which has moisture and can be slightly molded. According to Casagrande's Soil Classification System (ASTM) [21] it was defined as ML, inorganic silt and very fine sands with low plasticity. Besides laminated silt there can be less than 5% of rounded pebbles found, mainly ranging from 1 cm to 5 cm and rarely to 10 cm. Lacustrine chalk is covered by diamicton; main evidence can be found near to Hurja farm.

*Older alluvial sediments* can generally be found between Kred and Podbela up to 320 m above sea level as erosion patches around 40 m^2^ in size and are consequently not thicker than 5 m. They include conglomerate, but sands and gravel are more rare. First is clast supported conglomerate which is good sorted. The clasts, which range from 0.2 to 5 cm in size, are well rounded and consist mainly of carbonate and flyschoid fragments.

*Alluvial sediments* are located along former and actual steam channels of Nadiža River and Bela creek and in alluvial terraces of Kred polje (Figure 3(d)) and Podbela area. The volume ratio of sand and pebbles is 60 : 40. Fragments, which consist of carbonate and flyschoid formations, are well-rounded and moderately sorted. Thickness of alluvium deposits is estimated to be more than 10 m.

*Fine grained alluvial sediments* are deposited in Kred polje (Figure 3(d)) and Kobariško blato which represent a sedimentary basin between Mt. Stol and Mt. Matajur. The flat relief induces rare outcrops, whereas some exposures were seen in old melioration trenches. Some 4 m pits, which were excavated in the frame of geological investigations for planned wastewater treatment plant, were also examined. A particular sediment found in trenches and pits is characterised by silt of bluish grey colour. According to ASTM it is defined as ML, inorganic silt with low plasticity. On the foothills of Mt. Stol the formation is overlain by alluvial fan sediments.

*Alluvial fan sediments (proluvium)* (Figure 2(f)) are deposited by torrential creeks on the foothills of Mt. Stol from west of Staro Selo to Potoki (Figure 3(d)). In Podbela alluvial fan sediments are found as well. The formation consists of flyschoid and carbonate fragments, whereas the ratio depends on geological setting of hinterlands. Proluvium is poorly sorted since it consists of fragments generally ranging up to 10 cm, of which up to 40% is fine sand and silt. Boulders ranging up to 60 cm can also be observed. In addition, fragments are medium rounded. Thickness of formation is estimated on less than 20 m.

*Slope talus (gravel)* is deposited below steep slopes of Mt. Stol and Mt. Matajur. The lower boundary of the slope talus on the slope of Mt. Stol is increasing in elevation from east to west, whereas on the slope of Mt. Matajur it is decreasing in the same direction. Gravel consists of limestone and dolomite fragments of Late Triassic. They were distinguished by the reaction with dilute HCl. Besides gravel sized fragments, also significant amount of cobbles and some boulders can be found. Additionally patches of lithified slope tallus (gravel breccia), which have the same origin as unlithified, can be observed, but they are not presented in the map due to their negligible size and dispersion.

The described lithologies are presented on the detailed engineering geological map (Figure 4), which is the basis for the seismic microzonation of the studied area.

## 4. Intensity Increments Seismic Microzonation

**Figure 5:**
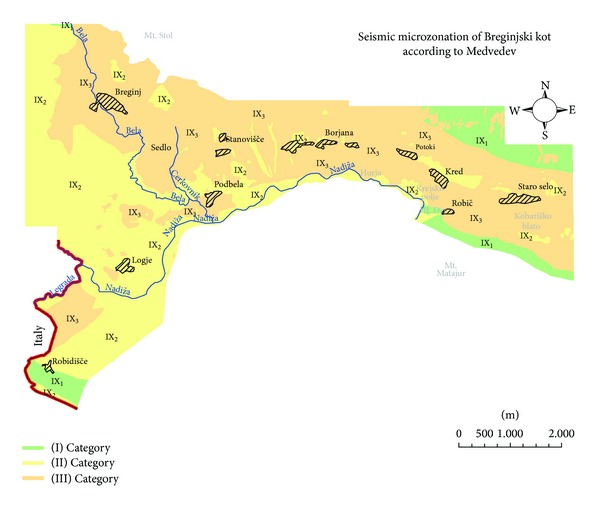
Intensity increments seismic microzonation of Breginjski kot according to the Medvedev method.

According to the old seismic hazard map of Slovenia for a 500-year return period [14] Breginjski kot is estimated to have IX MSK intensity. It is well-known that variations in local site characteristics have influence on observed earthquake intensity. Medvedev [3] proposed a classification that improves estimation on intensity considering engineering geological conditions. According to this classification, the soil is divided into three categories. The 2nd category corresponds to the medium stiff ground, which agrees with the given intensity on the seismological map [14], whereas for the 1st category an intensity decrease, and for the 3rd category an intensity increase is expected. According to this scheme Breginjski kot is subdivided into three categories (Table 3) and presented in the map (Figure 5). In addition, engineering geological conditions, topography, and soil saturation are given descriptively in the text below, according to Medvedev [3] and Mayer-Rosa and Jimenez [22].

*Category I* represented by Dachstein limestone and coral limestone which are in general seismically insensitive. But, due to limestone karstification some intensity increments may be expected also for these rocks. In addition, terrain subsidence is expected because of cave collapses. Severe earthquakes may also trigger rockfalls and rock slides which can endanger settlements or road facilities.

*Category II* is characterised by flyschoid formations, limestone breccia interbedded with shalestone, older alluvial sediments, and alluvial sediments. Besides them, diamictit and slope tallus (gravel breccia) are also within this category, but are not presented on the map because of negligible size and dispersion. Intensity increments may be significant due to the high- degree saturation of soil along Nadiža River or Bela creek and limestone breccia karstification. Severe earthquakes may also trigger rockfalls and rock slides which can endanger settlements or road facilities. Areas with increased thickness of weathered material are prone to landside occurrences. Many caves, abysses, and sinkholes are observed in the Robidišče plateau (Figure 3(a)), where the thickness of weathered material in general exceeds 5 m and can even reach 20 m in sinkholes. A local increment of intensity can be therefore expected.

*Category III* is represented by lacustrine chalk, fine grained alluvial sediments, alluvial fan sediments (proluvium), diamicton, and slope tallus (gravel). Intensity increments might be larger at contacts between different sediments, especially in Podbela, Krejsko polje, and Kobariško blato which are located on the foothills of Mt. Stol. Moreover, prominent topography changes like in Sedlo, which is situated in crest vicinity, can cause an additional intensity increment.

Since significant diversities in geological conditions and topography are observed in the studied area, which might cause an increase of intensity, five main reasons for site effects in different settlements of Breginjski kot are given in Table 4. Soft sediments should be considered as a complex set of depositional events of distinctive origin, hence different sediment layers exist, divided by a certain number of sediment contacts. Generally, the larger the number of contacts is, the larger the intensity increment is. In addition, prominent topography with steep slopes, sharp relief changes and abundant karst features are also reasons for an intensity increase.

Since 2008 seismic intensity is not used in Slovenia for building design anymore, but it is still important for seismological analysis and for civil protection. Broader applicability of the method is also in comparison with similar studies. The new seismic microzonation according to Medvedev method improves the older seismic microzonation [1] in many aspects: (a) mapping was performed on a larger scale, (b) lithologies descriptions are more precise and new interpretations are made and (c) map is digitalized. Moreover, the Bovec basin seismic microzonation [19] can also be compared to Breginjski kot due to geographic proximity and similar geological structures, though many different geotechnical and geophysical data were also acquired in the Bovec basin, as well as a study based on the microtremor method [23].

## 5. Eurocode 8 Seismic Microzonation

**Figure 6:**
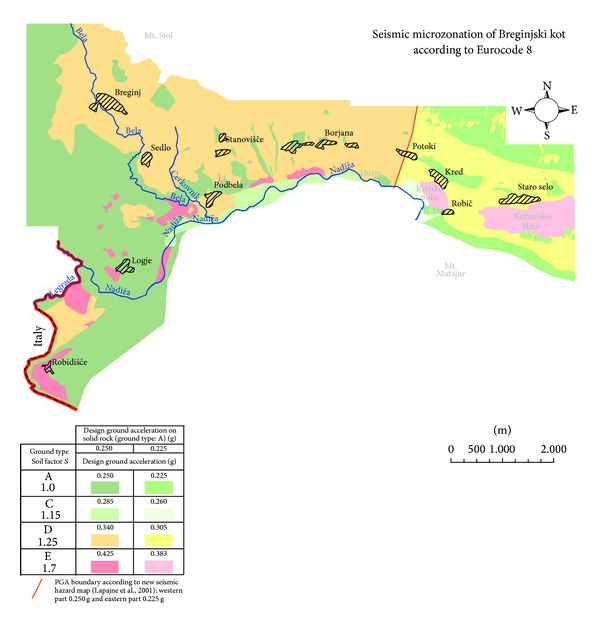
Eurocode 8 seismic microzonation of Breginjski kot.

The new seismic hazard map of Slovenia defines the seismic hazard with the peak ground acceleration. Furthermore, local ground conditions account for the additional influence on the seismic ground motion. In Europe ground types with different soil factors are usually determined according to the seismic standard Eurocode 8 [2] and National Annex [24]. According to the seismic hazard map for a 475-year return period [15], Breginjski kot is characterized in its larger western part by a design ground acceleration value for a rock site of 0.250 g and in the smaller easternmost part by 0.225 g. Ground types are classified on the basis of their geomechanical properties and weathered material thickness. Thus four different ground types, namely A, C, D, and E were assessed for the studied area (Figure 6).

*Class A* represents the least vulnerable ground type. It is characterised by limestone, limestone breccia interbedded with shalestone and flyschoid formation, and less than five meters of weathered material. According to the map (Figure 6) the concentration of class A is the largest in the westernmost part, though small patches are present throughout the area. Logje is the only settlement which is located entirely on class A ground. Only small parts of Robič, Potoki, and Borjana are lying on class A ground.

*Class C* is characterized by alluvial sediments, several tens of meters thick. The area which corresponds to the class C is situated along Nadiža River or Bela creek where no settlement is located.

*Class D* is characterized by diamicton, alluvial fan sediments, and slope tallus (gravel). This is the largest area in the map, which is mainly located on the slopes of Mt. Stol and Mt. Matajur. In addition it can be found in two patches between Sedlo and Robidišče. Due to its wide extent, most settlements belong to this class, for example, Staro Selo, Kred, Robič, Potoki, Borjana, Podbela, Sedlo, and Breginj.

*Class E* has the highest soil factor according to Eurocode 8. It comprises soil type C or D with a thickness varying from 5 m to 20 m underlain by a rock of type A. In addition, fine grained alluvial and lacustrine sediments belong to the latter type as a result of their poor geomechanical properties and heterogeneity. The area of class E includes Kobariško blato, Krejsko polje, Robidišče plateau, and some smaller patches along Nadiža valley. Robidišče is the only settlement which corresponds to the class E.

In addition to the upper classification some additional risks persist for each class, such as terrain subsidence in areas covered by limestone due to karst caves collapse and possibility for rockfalls. Final design acceleration values for each ground type are computed in accordance to the following expression:

(1) $$\begin{array}{c} {\text{DGA}}_{\text{F}}={\text{DGA}}_{\text{A}}\times S\times T_{\text{NCR}}, \end{array}$$

where final design ground acceleration (DGA_F_) corresponds to design ground acceleration on type A ground spectral acceleration multiplied by the soil factor (*S*) and the factor for the reference return period (*T*_NCR_). Topographic factor is neglected in this study but it should be taken into account for important structures according to Eurocode 8 [25]. Final design ground acceleration values are shown in Table 5. According to the National Annex [24] the soil factor for ground type E is 1.7, whereas in the original Eurocode 8 standard [2] it is 1.4; for all other ground types the soil factors are the same.

## 6. Discussion of Results

According to the engineering geological map of Breginjski kot, twelve different lithologies are presented and described in detail. Several of them are interpreted anew compared to previous studies (e.g., [18]).

Since typical glacial sediments are missing due to mass flows, alternative term diamicton for pseudoglacial sediments is suggested. Diamictit is also presented but only locally and in small patches. A recent study has shown that similar types of soft sediments or rocks are also identified in nearby Bovec basin [26]. Furthermore, gravel breccia was also observed in small patches similar to diamictit. Neither of them are presented in the engineering geological map but are only presented descriptively, because of their small size and sparse occurrence.

Fine grained alluvial sediments in Krejsko polje and Kobariško blato were deposited after Nadiža River had changed the current direction from the relatively wide Kobariško blato valley into the recent Nadiža valley, which is very narrow, with steep and rocky slopes [20]. Hence relatively large area was covered by swamplands and oxbow lakes where fine grained material was deposited and accumulated throughout Quaternary, although intense melioration in modern ages results in controlled river channel and thus in decreased sedimentation.

Because of detailed mapping, lithologies are defined more accurately and restricted in comparison with previous studies [20]. Therefore, their position and geometry can be better understood, thus contributing to the adequate estimation of the number of sediment contacts, their thicknesses, and abundance of karst phenomena, which may result in additional site effects (Table 4). Moreover, thicknesses of weathered material were estimated for each rock formation, because this also has influence on seismic effects.

A significantly large thickness of weathered material is located in the Robidišče plateau due to extreme weathering of shalestone which outcrops at the edge of the plateau. The Robidišče plateau is characterized by limestone breccia and coral limestone, which includes karst features like sinkholes where the thickness of weathered material generally exceeds 5 m. Moreover, the thickness of weathered material varies significantly, since in some areas bedrock outcrops are observed. Karst phenomena like caves and abysses can also contribute to an increase of seismic ground motion. According to the geological mapping, limestone breccia interbedded with shalestone is less subjected to karst feature occurrences or such features are scarce. In the settlements of Breginjski kot small thickness of weathered material was observed especially in Logje, where outcrops of rock formation are abundant. On the other hand, the outcrops in Borjana, Potoki, and Robič were limited, thus soft sediments are more abundant. Their cumulative thickness of less than 5 m is expected. However, it should be noted that geological characteristics of sediments and their thickness are estimated from the engineering geological mapping, whereas for a more detailed analysis systematic geotechnical drilling and geophysical investigations should be performed.

The damage to buildings in settlements of Breginjski due to the 1976 Friuli earthquake was unevenly distributed, although their distance from the epicentre does not vary significantly. For comparison, the soil classification is determined for each settlement of Breginjski kot according to both seismic microzonations. A comparison between intensities of the 1976 earthquake and soil classifications is given in Table 6.

Soil classification according to Medvedev method does not show a clear correlation of ground type to observed intensities. The main reason for this is that Medvedev soil classification does not take into account the thickness of soft sediments or weathered material. In addition it does not consider sufficiently engineering geological conditions which are annotated descriptively only. On the contrary, Eurocode 8 soil classification clearly shows a correlation between the ground type and observed intensities. This standard classifies ground types more precisely, also since it considers the thickness of soft sediments and weathered material. A better evaluation of local soil effect is therefore achieved. Based on the 1976 earthquake intensities, the settlements of Breginjski kot can be divided into two categories. The first group consists of settlements which show relatively low site effects like Borjana, Kred, Robič, Potoki, and Logje. They are located either on ground type A, or on the weathered material less than 5 m thick. The indicators of small weathered material thickness are frequent erosion windows. The second group consists of settlements which show relatively high site effects like Staro Selo, Sedlo, Breginj, Podbela, and Robidišče. They are located on soft sediments with considerable thickness. Additional causes of significant site effects in each particular settlement are described in Table 4.

## 7. Conclusions

This study represents a contribution to the evaluation of the seismic hazard in Breginjski kot, which is among the most endangered seismic areas in Slovenia. Two new seismic microzonations were prepared, because the previous seismic microzonation was based solely on the basic geological map and did not include supplementary field research, thus making it fairly inaccurate. In this study detailed engineering geological mapping of Breginjski kot in scale 1 : 5.000 was conducted. The results of mapping showed a diverse composition of sediments and rocks. Mapped units were described in detail and some of them are interpreted anew. Descriptions of mapped units also include the estimated thickness of soft sediments and weathered material. The results can be enhanced and validated by drilling and geophysical investigations, which is recommended especially in the settlements that were badly damaged during the 1976 Friuli earthquake. In addition, a microtremor method study would be appropriate to apply in the flatlands of Krejsko polje and Kobariško blato to determine soil resonance frequencies.

On the basis of engineering geological mapping, seismic microzonation was prepared according to the Medvedev method and the Eurocode 8 standard. According to the first classification the soil in Breginjski kot was divided into three categories, and according to the latter classified into categories A, C, D, and E. Two maps of seismic microzonation, based on both soil classifications, were made in the GIS platform and are therefore ready to use in urban planning. Seismic microzonation according to Eurocode 8 is more appropriate for the application, because it is in accordance with the modern seismic hazard standards and because it better correlates thickness of soft sediments or weathered material with site amplification. Moreover, ground types are classified more precisely, and thus the correlation with the geological map is better. The new seismic microzonation reveals the correlation of site effects and observed intensities. Sites located on softer ground experienced a higher degree of damage during the Friuli 1976 earthquake. Although the microzonation based on seismic intensities is not used in earthquake engineering anymore, it is still important for civil protection purposes and in seismological analysis. The new map improves the old microzonation and enables a comparison of geologically similar areas of Breginjski kot and the Bovec Basin.

## Figures and Tables

**Table 1:** Data for earthquakes with Mw ≥ 5.0 in the vicinity of Breginski kot (after [4, 7, 8]).

Date	Time (UTC)	Lat (°N)	Lon (°N)	Depth (km)	Mw	Region
06/05/1976	20:00	46.275	13.246	6.5	6.4	Friuli
09/05/1976	00:53	46.214	13.326	8.5	5.1	Friuli
11/05/1976	22:44	46.260	13.041	6.0	5.0	Friuli
11/09/1976	16:31	46.275	13.224	6.5	5.2	Friuli
11/09/1976	16:35	46.256	13.233	4.3	5.6	Friuli
15/09/1976	03:15	46.284	13.173	5.0	5.9	Friuli
15/09/1976	09:21	46.300	13.145	7.0	6.0	Friuli
16/09/1977	23:48	46.268	13.016	8.0	5.3	Friuli
12/04/1998	10:55	46.309	13.632	7.6	5.6	Krn Mts.
12/07/2004	13:04	46.310	13.620	11.0	5.2	Krn Mts.

**Table 2:** Intensities of 1976 earthquake in different settlements of Breginjski kot.

Settlement	Intensity EMS-98
Borjana	VI
Kred	VI
Robič	VI
Potoki	VI+ (MSK-78)
Logje	VI-VII
Staro Selo	VII
Sedlo	VII-VIII
Breginj	VIII
Podbela	VIII
Robidišče	VIII-IX

**Table 3:** Soil categories for seismic microzonation according to Medvedev method (after [3]).

Soil category	Soil type	Intensity increment according to seismic area		
		VII	VIII	IX
I	hard rocks	VI	VII	VIII
II	medium-hard rocks, lithified Quaternary sediments, alluvial sediments	VII	VIII	IX
III	unlithified Quaternary sediments	VIII	IX	X

**Table 4:** Main reasons for significant site effects in settlements of Breginjski kot.

Settlement	Main reasons for site effects				
	Prevailing lithology	No. of sediment contacts	Thickness to the basement rock	Topography	Karst features
Borjana	Diamicton	1	5–10 or more	Steep-moderate slopes	no
Kred	Proluvium	2	<10 m	Flat	no
Robič	Gravel and limestone	2	<5 m	Flat	no
Potoki	Proluvium and flyschoid f.	1	0–10 m or more	moderate slopes	no
Logje	Flyschoid f.	0*	<2 m	slight slopes-flat	no
Staro Selo	Proluvium	3	>20 m	slight slopes-flat	no
Sedlo	Diamicton	1	10–20 m or more	slight slopes, sharp relief change	no
Breginj	Diamicton	1	>20 m	slight slopes-flat	no
Podbela	Proluvium	2	>20 m	flat	no
Robidišče	Limestone and limestone breccia	0*	>5 m	flat	yes

*The thickness of weathered material is estimated in text.

**Table 5:** Final design ground acceleration values for each soil type in Breginjski kot.

Soil type	*S*	*T*_ NCR_	DGA_A_ [g]	
			0.250	0.225
			DGA_F_ [g]	
A	1.0	475 years	0.250	0.225
C	1.15		0.285	0.260
D	1.35	1.00	0.340	0.305
E	1.70		0.425	0.383

A	1.0	1000 years	0.315	0.280
C	1.15		0.360	0.325
D	1.35	1.25	0.420	0.380
E	1.70		0.531	0.478

**Table 6:** Intensities of 1976 earthquake in different settlements of Breginjski kot and soil classification after Medvedev and Eurocode 8 from both seismic microzonation maps.

Settlement	Intensity EMS-98	Soil classification	
		After Medvedev	After Eurocode 8
Borjana	VI	III, II	D, A
Kred	VI	III	D
Robič	VI	III, I	D, A
Potoki	VI+ (MSK-78)	III, II	D, A
Logje	VI-VII	II	A
Staro Selo	VII	III	D
Sedlo	VII-VIII	III	D
Breginj	VIII	III	D
Podbela	VIII	III	D
Robidišče	VIII-IX	II	E

## References

[1] Vidrih R, Godec M, Lapajne J (1991). *Earthquake Risk in Slovenia*.

[2] CEN (2004). *Eurocode 8—Design of Structures for Earthquake Resistance, Part 1: General Rules, Seismic Actions and Rules for Buildings, European Standard, EN, 1998-1: 2004 (E), Stage 64*.

[3] Medvedev SV (1965). *Engineering Seismology*.

[4] Perniola B, Bressan G, Pondrelli S (2004). Changes in failure stress and stress transfer during the 1976–77 Friuli earthquake sequence. *Geophysical Journal International*.

[5] Carulli GB, Slejko D (2005). The 1976 Friuli (NE Italy) earthquake. *Giornale di Geologia Applicata*.

[6] Ribarič V (1980). Earthquakes in Friuli in 1976 and the short history of seismicity at the margin of Eastern Alps. *Potresni Zbornik*.

[7] Zupančič P, Cecić I, Gosar A, Placer L, Poljak M, Živčić M (2001). The earthquake of 12 April 1998 in the Krn Mountains (Upper Soča valley, Slovenia) and its seismotectonic characteristics. *Geologija*.

[8] Cecić I, Živčić M, Jesenko T, Kolar J (2006). Earthquakes in Slovenia in 2004. *Potresi V Letu 2004*.

[9] Bajc J, Aoudia A, Saraò A, Suhadolc P (2001). The 1998 Bovec-Krn mountain (Slovenia) earthquake sequence. *Geophysical Research Letters*.

[10] Gosar A (2010). Site effects and soil-structure resonance study in the Kobarid basin (NW Slovenia) using microtremors. *Natural Hazards and Earth System Sciences*.

[11] Kastelic V, Vrabec M, Cunningham D, Gosar A (2008). Neo-Alpine structural evolution and present-day tectonic activity of the eastern Southern Alps: the case of the Ravne Fault, NW Slovenia. *Journal of Structural Geology*.

[12] Fitzko F, Suhadolc P, Aoudia A, Panza GF (2005). Constraints on the location and mechanism of the 1511 Western-Slovenia earthquake from active tectonics and modeling of macroseismic data. *Tectonophysics*.

[13] Camassi R, Caracciolo CH, Castelli V, Slejko D (2011). The 1511 Eastern Alps earthquakes: a critical update and comparison of existing macroseismic datasets. *Journal of Seismology*.

[14] Ribarič V (1987). *Seismological Map of Slovenia for 500 Years Return Period*.

[15] Lapajne J, Šket-Motnikar B, Zupančič P (2001). Design ground acceleration map of Slovenia. *Potresi V Letu 1999*.

[16] Ambraseys N, Smit P, Sigbjornsson R, Suhadolc P, Margaris B Internet-Site for European Strong-Motion Data. http://www.isesd.hi.is/ESD_Local/frameset.htm.

[17] Šket-Motnikar B, Prosen T (2006). Accelerations in Posočje on July 12 2004. *Potresi V Letu 2004*.

[18] Buser S (1986). *Basic Geological Map of Yugoslavia 1: 100. 000—Sheet Tolmin and Videm*.

[19] Ribičič M, Vidrih R, Godec M (2000). Seismogeological and geotechnical conditions of buildings in Upper Soča Territory, Slovenia. *Geologija*.

[20] Buser S (1986). *Basic Geological Map of Yugoslavia 1: 100. 000—Sheet Tolmin and Videm Explanatory Text*.

[21] Casagrande A (1948). Classification and identification of soils. *Transactions of the American Society of Civil Engineers*.

[22] Mayer-Rosa D, Jimenez MJ (2000). *Seismic Zoning: State-of-the-Art and Recommendations for Switzerland*.

[23] Gosar A (2007). Microtremor HVSR study for assessing site effects in the Bovec basin (NW Slovenia) related to 1998 Mw5.6 and 2004 Mw5.2 earthquakes. *Engineering Geology*.

[24] SIST (2004). *Slovenian Standard SIST EN, 1998-1 Eurocode 8: Design of Structures for Earthquake Resistance—Part 1: General Rules, Seismic Actions and Rules for Buildings—National Annex*.

[25] CEN (2004). *Eurocode 8—Design of Structures For Earthquake Resistance, Part 5: Foundations, Retaining Structures and Geotechnical Aspects, European Standard, EN, 1998-5: 2004 (E), Stage 64*.

[26] Bavec M (2002). New temporal, and genetic determinations of some late Quaternary sediments in the Bovec basin and its surroundings (NW Slovenia). *Geologija*.

